# Membrane-Modified Metal Triazole Complexes for the Electrocatalytic Reduction of Oxygen and Carbon Dioxide

**DOI:** 10.3389/fchem.2018.00543

**Published:** 2018-11-06

**Authors:** Skye N. Supakul, Christopher J. Barile

**Affiliations:** Department of Chemistry, University of Nevada, Reno, NV, United States

**Keywords:** electrocatalysis, carbon dioxide reduction, oxygen reduction, self-assembled monolayer, flip-flop diffusion, lipid monolayer

## Abstract

In this manuscript, an electrochemical architecture is designed that controls the kinetics of proton transfer to metal triazole complexes for electrocatalytic O_2_ and CO_2_ reduction. Self-assembled monolayers of these catalysts are attached to a glassy carbon electrode and covered with a lipid monolayer containing proton carriers, which acts as a proton-permeable membrane. The O_2_ reduction voltammograms on carbon are similar to those obtained on membrane-modified Au electrodes, which through the control of proton transfer rates, can be used to improve the selectivity of O_2_ reduction. The improved voltage stability of the carbon platforms allows for the investigation of a CO_2_ reduction catalyst inside a membrane. By controlling proton transfer kinetics across the lipid membrane, it is found that the relative rates of H_2_, CO, and HCOOH production can be modulated. It is envisioned that the use of these membrane-modified carbon electrodes will aid in understanding catalytic reactions involving the transfer of multiple protons and electrons.

## Introduction

The electrocatalysis of small molecules is important in a wide range of renewable energy devices (Duan et al., 2015; Zeng and Li, 2015; Zhang et al., 2017). Many of these conversion processes involve multiple proton and electron transfer steps (Mayer, 2004; Huynh and Meyer, 2007; Dai et al., 2015). For example, the electrocatalytic reduction of O_2_ to water, which occurs in the cathode of fuel cells, requires the transfer of four protons and four electrons (Gewirth and Thorum, 2010). Similarly, the CO_2_ reduction reaction, which can produce sustainable fuels, also requires multiple proton and electron transfer steps (Hori et al., 1985; Hori, 2008). These two reactions, along with most others requiring multiproton and multielectron transfer, are therefore mechanistically complex, both in terms of the reaction intermediates and in the range of products that are ultimately generated.

In both CO_2_ reduction and the O_2_ reduction reaction (ORR), the dynamics of proton transfer to catalytic sites are instrumental in dictating catalyst selectivity and performance (Hammes-Schiffer and Soudackov, 2008; Hammes-Schiffer, 2009). For small molecule electrocatalysts, several approaches have been used to interrogate the effect of proton transfer on catalysis. The most common methodology is to synthesize a group of ligands with pendant proton relays, which tune proton availability to a metal-centered catalytic core (Sjödin et al., 2005; Rosenthal and Nocera, 2007; Wenger, 2013). However, the incorporation of these proton relays often changes the steric and electronic environment of the catalyst, which in turn alters its redox properties (McCrory et al., 2007; Thorseth et al., 2013). An alternative strategy is to bury electrocatalysts inside lipid membranes with embedded proton carriers (Hosseini et al., 2011). This approach allows for proton transfer dynamics to the catalyst to be controlled without changing the molecular identity of the catalyst (Barile et al., 2014). These electrode platforms consist of a thiol-based self-assembled monolayer (SAM) on Au electrodes that are covered by a lipid monolayer appended via Van der Waals interactions (Tse et al., 2015).

Previous results demonstrate that the incorporation of alkyl proton carriers inside the lipid layer of these electrodes can be used to control the kinetics of proton transfer to catalysts (Tse et al., 2016). In particular, the selectivity of a Cu triazole ORR catalyst can be improved using this platform such that adequately tuned proton transfer rates cause water to be the sole product generated. Unfortunately, the extension of membrane-modified thiol-based Au SAMs to other catalytic systems is limited by their narrow electrochemical stability. Thiol-based Au SAMs are not stable at highly negative potentials (Srisombat et al., 2011), which means they cannot be used to study reduction reactions with high overpotentials such as CO_2_ reduction.

In this manuscript, membrane-modified carbon electrodes are designed that allow for proton transfer dynamics to electrocatalysts to be controlled and that exhibit greater electrochemical stability than their Au counterparts. The architecture developed here enables the interrogation of the membrane-modified ORR, and also the study of reactions such as the CO_2_ reduction reaction, which occurs at high overpotentials.

## Materials and methodology

Chemicals were obtained from commercial sources and were not subjected to additional purification. 1,2-dimyristoyl-*sn*-glycero-3-phosphocholine (DMPC) lipid was obtained from Avanti Polar Lipids, and the proton carriers dodecylboronic acid (DBA) and mono-n-dodecylphosphate (MDP) were obtained from Alfa Aesar. Triazole ligands were synthesized following a literature procedure (Li et al., 2015). For experiments under controlled temperatures, the temperature was maintained within 3 degrees of the reported value. Electrochemical analysis and attachments were conducted using a VSP-300 Biologic Potentiostat utilizing a three-electrode arrangement consisting of a leakless Ag/AgCl (3M KCl, eDaq, Inc.) reference electrode that is stable in both aqueous and ethanolic solutions, a Pt wire counter electrode, and a Au or glassy carbon working electrode. Previously reported procedures for experiments on Au electrodes were followed (Tse et al., 2016).

For experiments with carbon, a 5 mm diameter glassy carbon electrode was used as the working electrode. The working electrode was cleaned before each experiment by rinsing the electrode surface using de-ionized water, followed by manual polishing with 0.3 μm alumina particles suspended in de-ionized water on a polishing pad for approximately 6 min. After polishing, the electrode was sonicated in de-ionized water, followed by sonication in acetone, and finally sonication in isopropyl alcohol for 3 min each before being dried under a stream of air. For the electrochemical attachment of the amino-terminated triazole onto the glassy carbon electrode, cyclic voltammograms were conducted with the cleaned electrode in a 10 mL ethanolic solution containing 5 mM amino-terminated triazole and 100 mM LiClO_4_ from a potential of 2 V to −0.01 V at a scan rate of 10 mV/s for 10 cycles. Following the attachment, the electrode was sonicated in pH 7 potassium phosphate buffer for 10 min to remove excess, unattached triazole molecules. After sonication, the amino-terminated triazole surface was immersed in a 10 mL de-ionized water solution containing 10 mM CuSO_4_ or 10 mM AgNO_3_ for 1 hr to form the Cu-triazole or Ag-triazole complex, respectively. For the attachment of the lipid membrane containing a proton carrier, the glassy carbon electrode modified with the Cu-triazole complex was immersed in a 1 mL CHCl_3_ solution containing 7.4 mM DMPC and 5.6 mM proton carrier for 20 s followed by a brief submersion into 3 mL de-ionized water containing 100 mM KCl until excess CHCl_3_ solution separated away from the electrode surface. Finally, the membrane-modified electrode was rinsed with pH 7 phosphate buffer before electrochemical analyses were performed.

To test the catalytic activity of the membrane-modified glassy carbon electrode, O_2_ reduction and CO_2_ reduction reactions were performed. A pH 7 phosphate buffer solution was sparged with air or CO_2_ for a minimum of 20 min to ensure the solution was saturated with the specific gas. Electrocatalytic activity was evaluated using linear sweep voltammetry from 0.3 V to −0.7 V for O_2_ reduction or 0.3 V to −2.0 V for CO_2_ reduction at a scan rate of 10 mV/s. A blocking test to assess the integrity of the membrane-covered electrode was performed after each reduction reaction using a CV from 0.5 V to −0.5 V at a scan rate of 50 mV/s in a de-ionized water solution containing 1.5 mM K_3_Fe(CN)_6_ and 100 mM NaCl. The products of the CO_2_ reduction reaction were identified using protocols modified from the literature (Tornow et al., 2012). ^1^H NMR spectroscopy was used to quantify HCOOH production. ^1^H NMR spectra were obtained using a Varian 400 MHz NMR Spectrometer in the Shared Instrument Laboratory (SIL) in the Department of Chemistry at the University of Nevada, Reno (UNR). CO production was quantified using a colorimetric assay based on K_2_Pd(SO_3_)_2_ (Lin et al., 2018). GC-MS (Agilent 7890A) was used to identify any possible >2 e^−^ CO_2_ reduction products such as C_2_H_4_ and CH_4_.

## Results and discussion

### Ligand design

**Figure 1:**
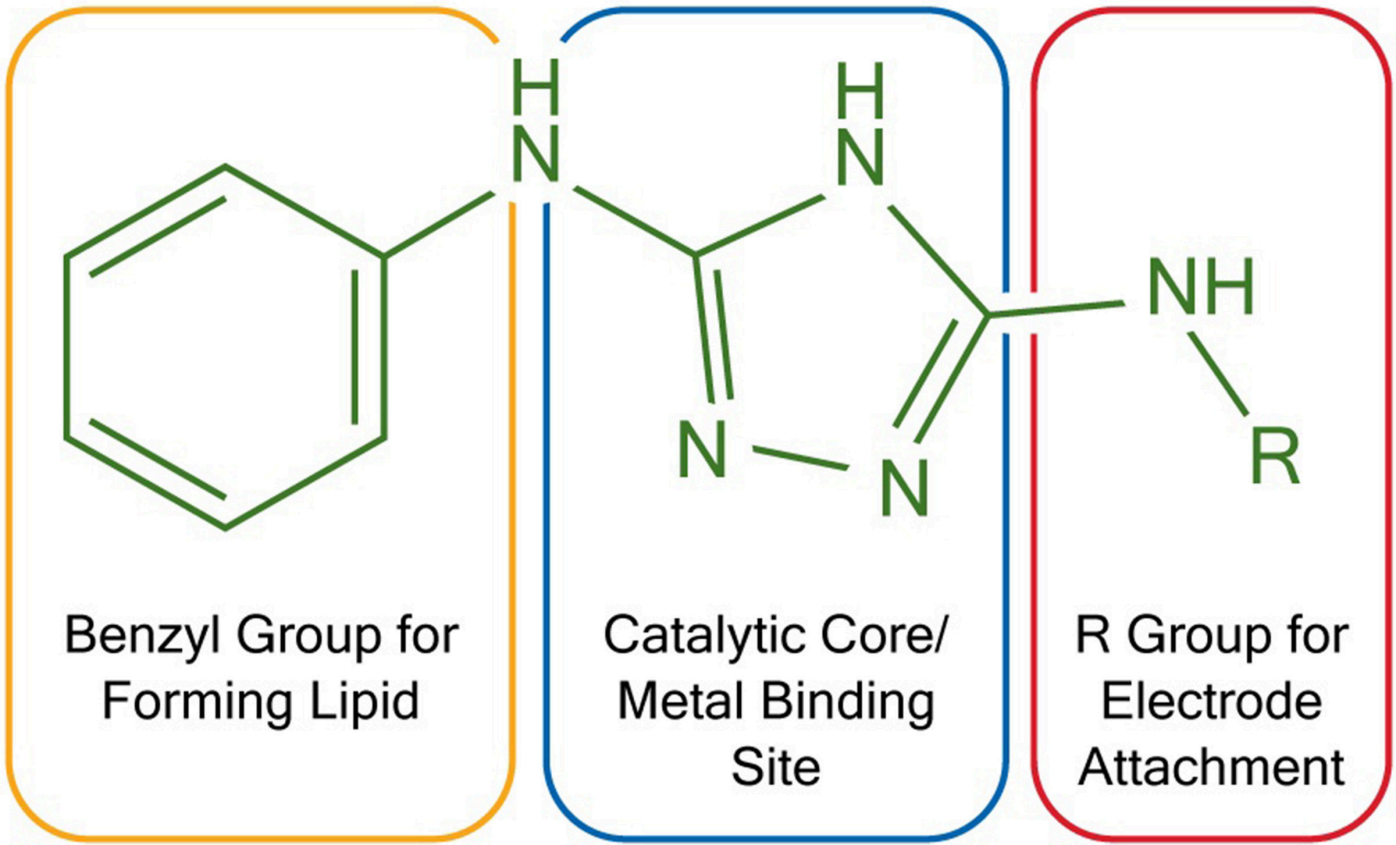
Functional and structural features of two triazoles used in electrocatalysis. *R* = –(CH_2_)_6_SH for Au electrodes and *R* = –H for carbon electrodes.

To construct membrane-modified electrodes for electrocatalysis, two triazole molecules were first synthesized following literature protocols (Li et al., 2015). The structure of these molecules can be divided into three different sections, each with a specific function. First, the molecules contain a diaminotriazole core (Figure 1, blue), which upon metal binding serves as the active electrocatalytic site. This catalyst core was studied because the dinuclear Cu complex of 3,5-diamino-1,2,4-triazole has previously been demonstrated to be an efficient and stable ORR catalyst (Thorum et al., 2009). Second, the molecules contain either an alkyl thiol or a primary amine arm (Figure 1, red). These functional groups allow the molecules to be covalently attached to either Au or carbon electrodes, respectively. Lastly, both molecules contain a hydrophobic benzyl group (Figure 1, orange) so that lipid layers can be appended on top of the catalysts via Van der Waals interactions.

### Electrocatalytic O_2_ reduction on membrane-modified au electrodes

**Figure 2:**
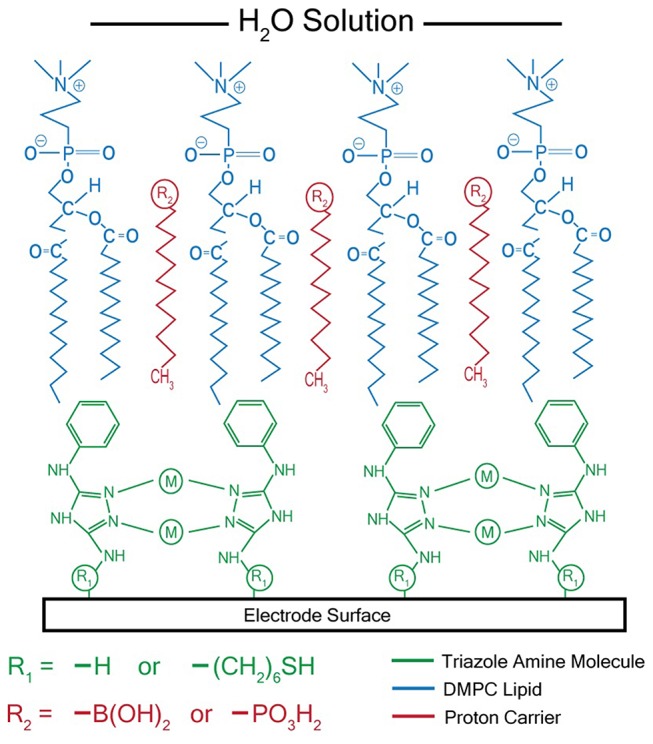
Schematic of membrane-modified electrode consisting of a metal triazole catalyst (green) and a lipid monolayer (blue) with proton carrier (red). M = Cu^2+^ or Ag^+^ for catalysts that reduce O_2_ or CO_2_, respectively.

**Figure 3:**
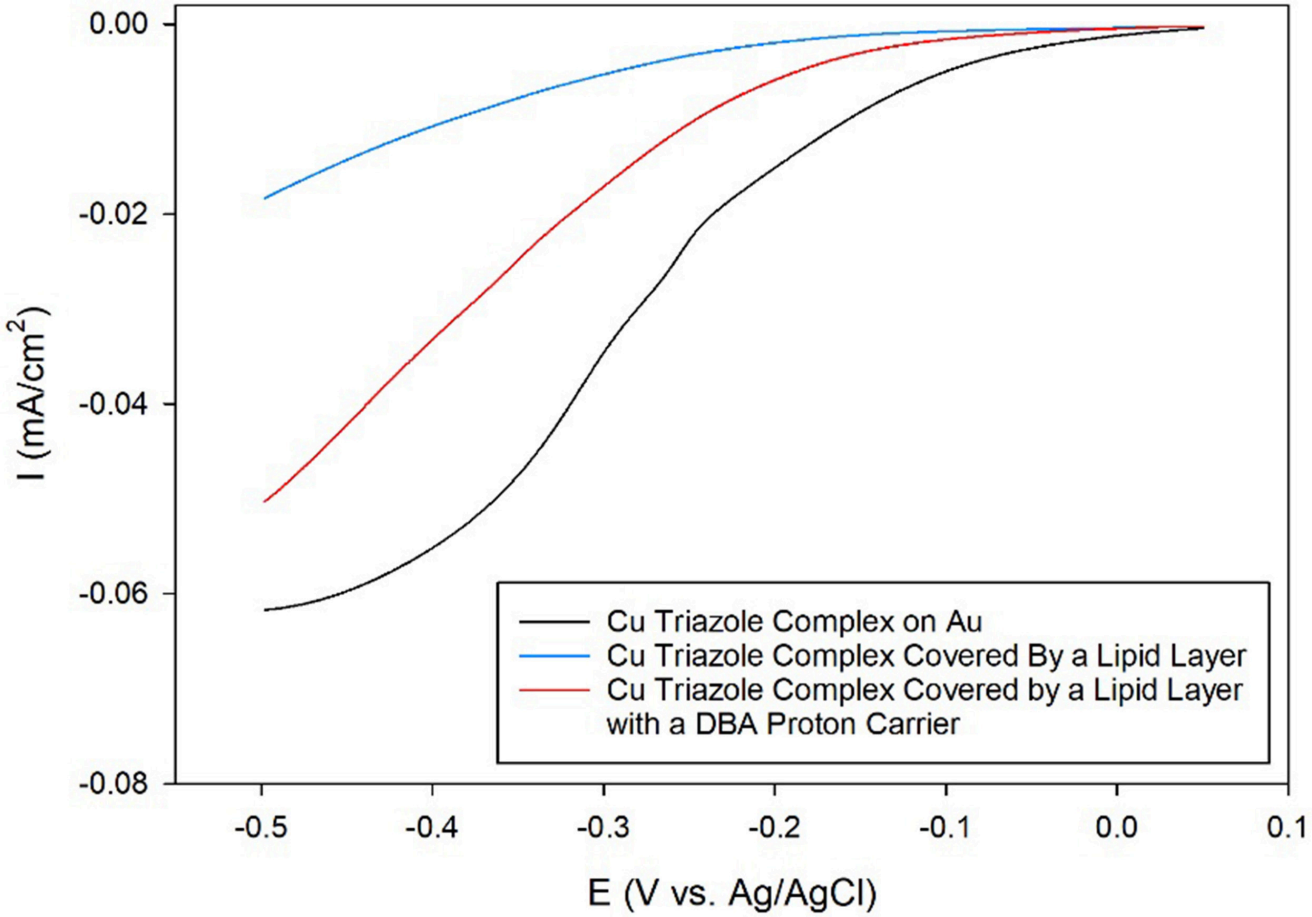
Linear sweep voltammograms of O_2_ reduction by a Au electrode modified with the Cu complex of the thiol-terminated triazole (black line) covered by a lipid membrane (blue line) with DBA proton carrier (red line) in pH 7 phosphate buffer at a scan rate of 10 mV/s.

After synthesizing these triazole ligands, the electrocatalytic activity of the Cu triazole complex on Au electrodes was first analyzed with and without lipid membranes. First, the Cu catalyst was attached to Au electrodes by forming a SAM of the thiol-modified triazole and subsequently immersing the SAM in a solution of CuSO_4_ (Figure 2, green, R_1_ = – (CH_2_)_6_SH). A linear sweep voltammogram (LSV) of the Cu catalyst in air-saturated pH 7 phosphate buffer displays an ORR onset potential of about 0 V and a diffusion-limited peak current density of about −0.062 mA/cm^2^ (Figure 3, black line). Upon covering the catalyst with a lipid monolayer (Figure 2, blue, R_1_ = – (CH_2_)_6_SH), the activity of the catalyst diminishes substantially due to slow proton transport through the hydrophobic lipid membrane (Barile et al., 2014; Figure 3, blue line). Strikingly, the incorporation of dodecylboronic acid (DBA) into the lipid layer (Figure 2, red, R_1_ = – (CH_2_)_6_SH) revives much of the catalytic activity (Figure 3, red line). These observations on Au match previous results and as discussed (Tse et al., 2016), the presence of lipid-bound proton carriers accelerates proton transfer to the ORR catalyst. In particular, the proton carrier enhances the current of the ORR, but does not significantly change the ORR onset potential as compared to the lipid-only case (Hosseini et al., 2011).

The enhancement of current elicited by the proton carrier is due to a change in the ORR mechanism as demonstrated in previous work (Tse et al., 2016; Gautam et al., 2018). In the presence of lipid layer without proton carrier, the hydrophobic nature of the lipid impedes proton transfer to the catalyst, which causes the ORR to proceed primarily via a 1 e^−^ pathway to yield superoxide. With the incorporation of proton carrier, the proton transfer rate to the catalyst is increased, which favors the 4 e^−^ reduction of O_2_ to water. In the absence of a lipid layer, the Cu catalyst reduces O_2_ by both 2 e^−^ and 4 e^−^ pathways to produce a mixture of H_2_O_2_ and water. The mechanistic details for all of these electrochemical environments are illustrated in Figure S1.

### Electrocatalytic O_2_ reduction on membrane-modified glassy carbon electrodes

**Figure 4:**
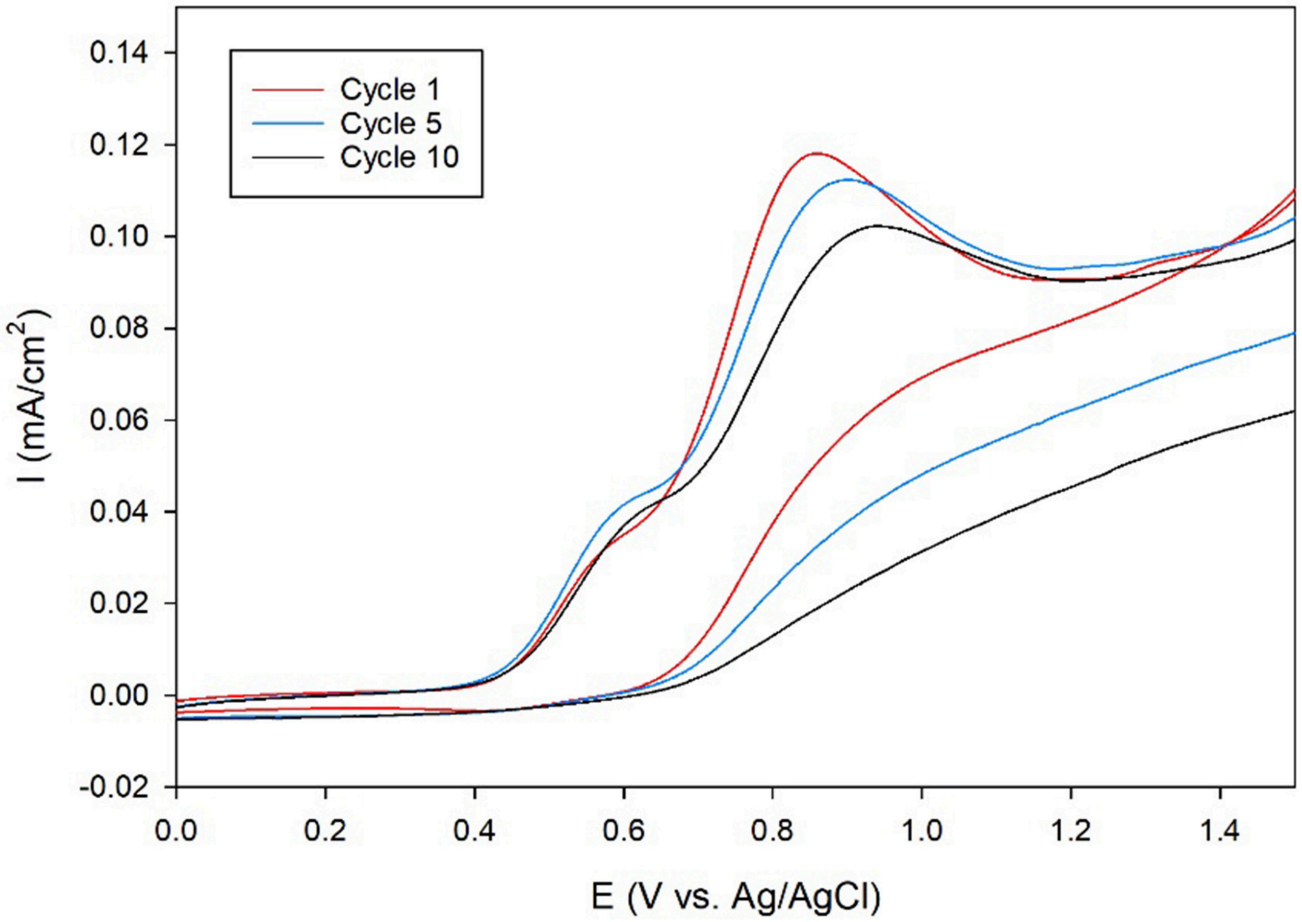
Cyclic voltammogram cycles 1 (red line), 5 (blue line), and 10 (black line) of a glassy carbon electrode in an ethanolic solution of 5 mM amino-terminated triazole and 100 mM LiClO_4_ at a scan rate of 10 mV/s.

Next, the ORR catalytic activity on carbon electrodes was analyzed since carbon is more durable and inexpensive than Au, making it the electrode of choice for commercial fuel cells. Toward this end, membrane-modified glassy carbon electrodes were designed. First, the amino-terminated triazole was covalently attached to the electrode surface through the oxidation of the primary amine group using cyclic voltammetry (CV). The CVs recorded during the attachment process display anodic peaks at around 0.8–0.9 V, which indicate that the amine is oxidized at the carbon surface (Figure 4). These results are similar to previous studies, which use CV to electrochemically attach primary alkyl amines to carbon electrodes (Deinhammer et al., 1994).

**Figure 5:**
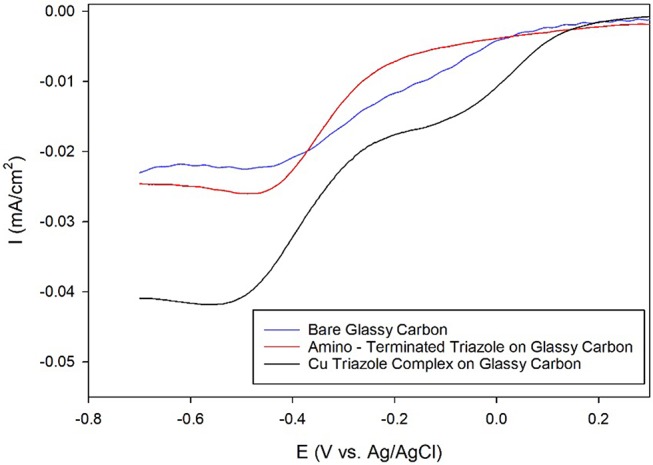
Linear sweep voltammograms of O_2_ reduction by a glassy carbon electrode (blue line) modified with the amino-terminated triazole (red line) and the Cu complex of the amino-terminated triazole (black line) in pH 7 phosphate buffer at a scan rate of 10 mV/s.

After electrochemical attachment of the amino-terminated triazole to the carbon electrode, the Cu-triazole complex was formed by soaking the electrode in a solution of CuSO_4_ (Figure 2, green, R_1_ = –H). The presence of a Cu(I)/Cu(II) redox couple in a CV of the Cu-modified electrode indicates that Cu was successfully incorporated into the electrode architecture (Figure S2). The Cu complex catalyzes the reduction of O_2_ with an onset potential of about +0.1 V and a diffusion-limited current density of about −0.04 mA/cm^2^ (Figure 5, black line). The onset potential for this catalyst is fairly similar to what is observed for O_2_ reduction by the Cu triazole complex immobilized on a Au electrode (Figure 3, black line). LSVs of O_2_ reduction by a bare glassy carbon electrode and an electrode modified with triazole in the absence of Cu exhibit more negative onset potentials and less diffusion-limited current as compared to catalysis by the Cu triazole complex (Figure 5, blue and red lines, respectively). These experiments indicate that the Cu triazole complex is a more active ORR catalyst than the controls.

**Figure 6:**
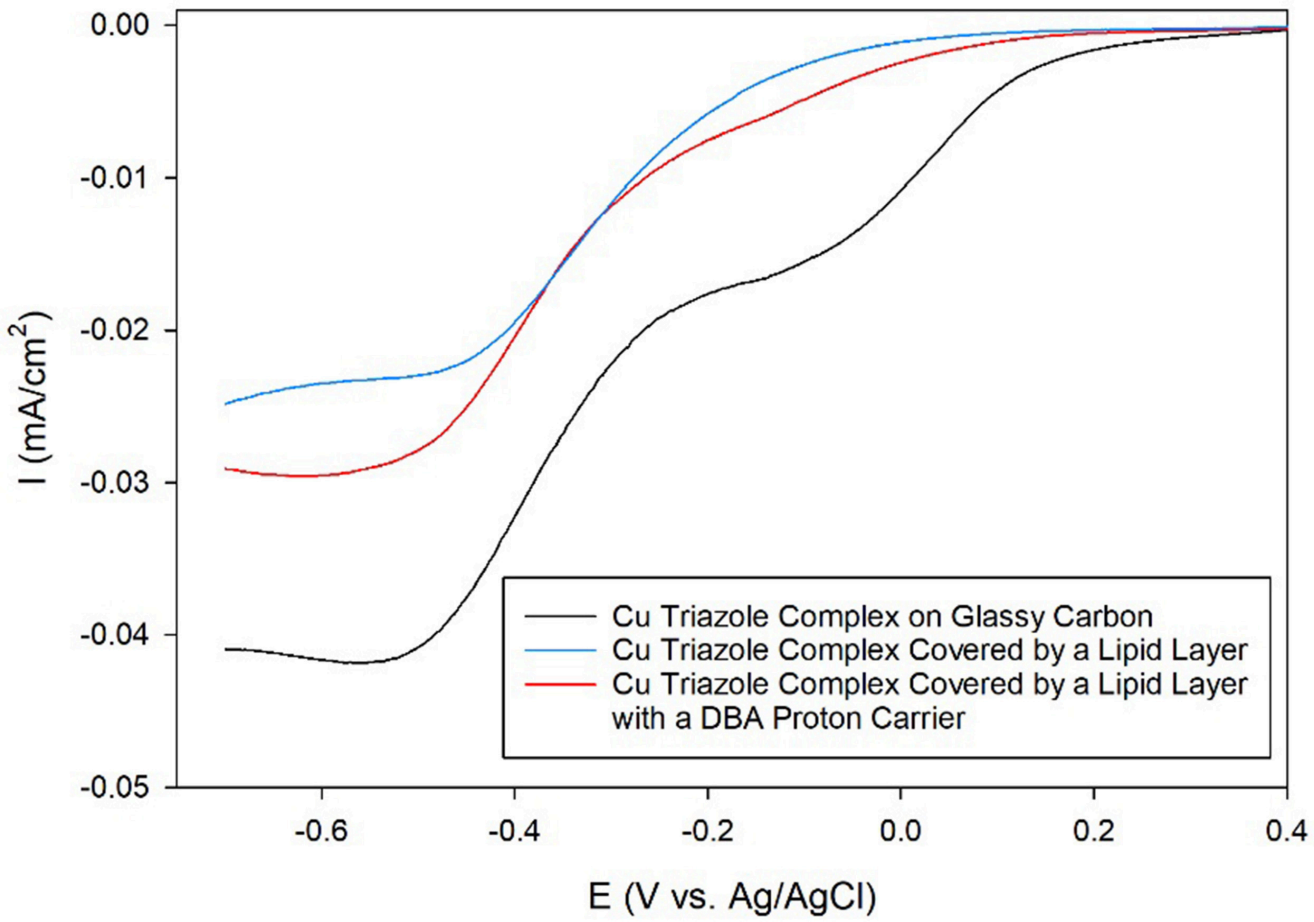
Linear sweep voltammograms of O_2_ reduction by a glassy carbon electrode modified with the Cu complex of the amino-terminated triazole (black line) covered by a lipid membrane (blue line) with DBA proton carrier (red line) in pH 7 phosphate buffer at a scan rate of 10 mV/s.

Having established the electrocatalytic activity of the Cu triazole complex on a carbon electrode, the surface was next modified with a lipid membrane to control proton transfer to the catalyst. The lipid membrane was formed by soaking the electrode in a solution containing DMPC using a method adapted from a previously reported procedure (Han et al., 2003; Figure 2, blue, R_1_ = –H). The incorporation of a lipid layer on top of the Cu catalyst shifts the onset potential for the ORR significantly negative and also decreases the diffusion-limited current density (Figure 6, blue line). This result indicates that the ORR is inhibited by the presence of the lipid layer due to impeded proton transfer to the catalyst by the hydrophobic membrane in a manner similar to the lipid-covered catalyst on Au. The addition of DBA, a boronic acid proton carrier, to the lipid layer (Figure 2, red, R_1_ = –H) increases the ORR diffusion-limited current density, but does not significantly alter the ORR onset potential (compare Figure 6, red line to blue line). The presence of a proton carrier accelerates proton transfer to the Cu catalyst, which results in the increased catalytic current density observed. The finding that the proton carrier enhances the current of the ORR, but does not significantly change the ORR onset potential as compared to the lipid-only case, suggests that the proton carrier increases the kinetics of the ORR without significantly affecting the reaction thermodynamics. Taken together, these results indicate that membrane-modified electrodes can be successfully formed on glassy carbon substrates and that the general electrochemical behavior of these systems is similar to those constructed on Au.

To assess the integrity of the lipid layer during the ORR, blocking experiments were performed using K_3_Fe(CN)_6_ in bulk solution after the ORR as described in other systems (Barile et al., 2016). A decrease in the current density observed from the Fe(II)/Fe(III) redox couple using lipid-modified electrodes as compared to membrane-free electrodes indicates that the lipid layer remains intact during the ORR process (Figure S3). In fact, the blocking data show that the Fe(II)/Fe(III) redox couple is less pronounced when the proton carrier is incorporated in the lipid layer (Figure S3, red line) as compared to lipid only case (Figure S3, blue line). These results demonstrate that the incorporation of the proton carrier into the lipid membrane does not adversely affect the integrity of the lipid layer and instead actually enhances lipid formation. Therefore, the increase in ORR current density elicited by the proton carrier is not caused by a disruption in the integrity of the lipid membrane. Instead, the proton carrier causes an increase in the electrocatalytic O_2_ reduction current density by the Cu catalyst because it accelerates the proton transfer rate to the catalyst. The origin of this current enhancement was further confirmed by averaging results obtained across eight experimental trials (Figure S4). This analysis demonstrates that the proton carrier enhances the kinetics of the ORR even when taking into account the integrity of the lipid layer as determined from subsequent blocking experiments.

### Electrocatalytic CO_2_ reduction on membrane-modified glassy carbon electrodes

Electrocatalytic CO_2_ reduction typically occurs at high overpotentials (Qiao et al., 2014). Hence, it is difficult to study CO_2_ reduction using membrane-modified Au electrodes because Au SAMs degrade at potentials more negative than about −0.5 V (Srisombat et al., 2011). To overcome this issue, a CO_2_ reduction catalyst was prepared on a membrane-modified glassy carbon electrode because carbon electrodes are more electrochemically stable. Furthermore, the majority of previous studies of CO_2_ reduction catalysts on carbon electrodes utilize a binder, most commonly Nafion, to adhere the catalyst to the electrode (Tornow et al., 2012; Thorson et al., 2013; Weng et al., 2018). Inks containing carbon, Nafion, and the catalyst are usually dropcast on a glassy carbon electrode to form a porous multilayer catalyst structure that is useful for practical high current density devices, but complicates catalyst surface structure and hinders fundamental electrochemical analysis.

In contrast to a multilayer architecture, we electrochemically attach a monolayer of catalyst to glassy carbon electrodes that do not require the use of a binder. This method of surface modification allows for a more direct assessment of the activity of molecular CO_2_ catalysts. Moreover, binders such as Nafion dramatically alter the proton transfer rates to embedded catalysts. The binder-free system devised here enables us to systematically analyze the effect of proton transfer on catalyst performance. In a manner similar to the previously described Cu triazole ORR catalyst, the kinetics of proton transfer to a CO_2_ reduction catalyst can be tuned by covering the catalyst with a lipid monolayer.

**Figure 7:**
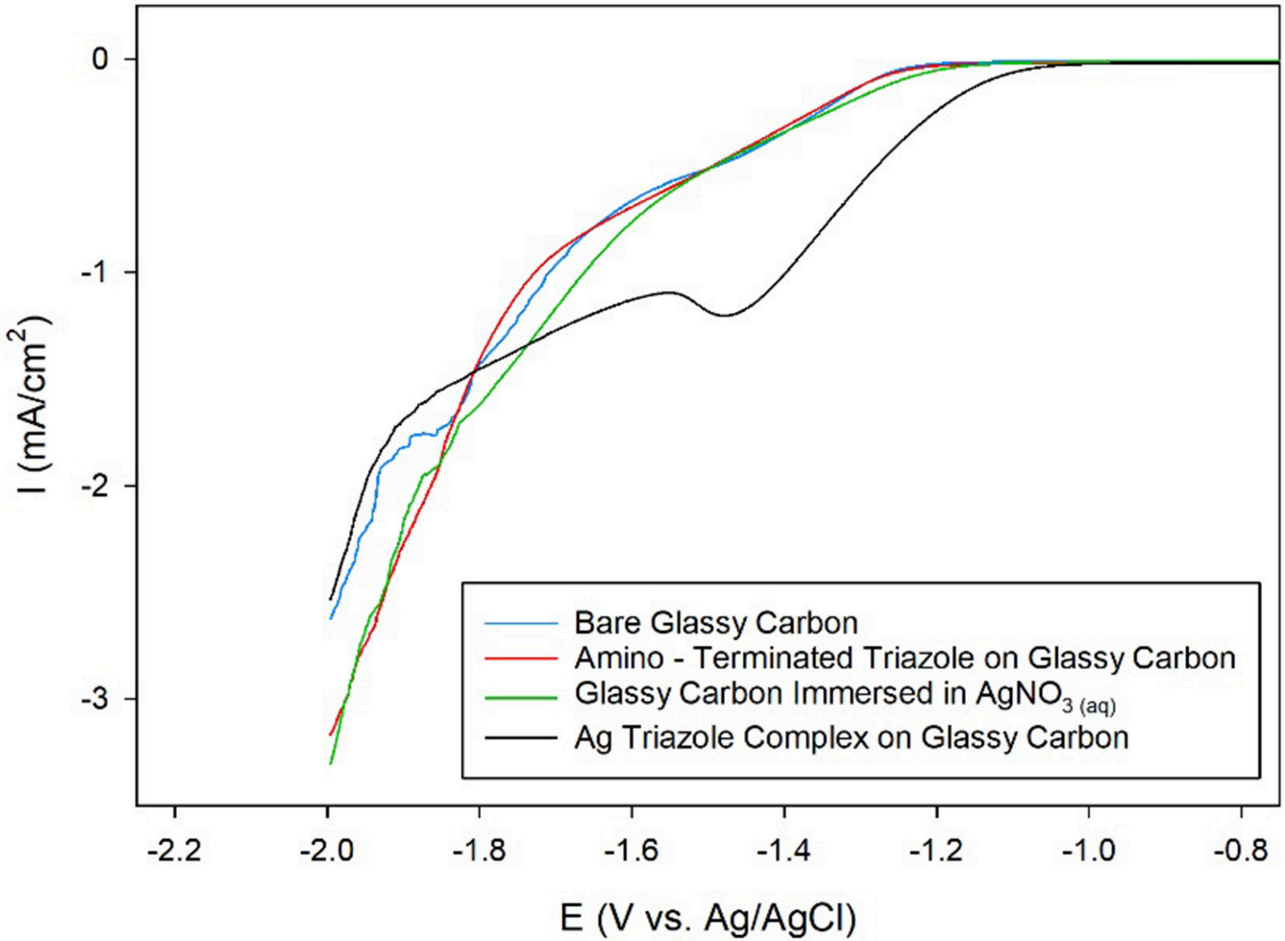
Linear sweep voltammograms of CO_2_ reduction by a glassy carbon electrode (blue line) modified with the amino-terminated triazole (red line) and the Ag complex of the amino-terminated triazole (black line) in pH 7 phosphate buffer at a scan rate of 10 mV/s. CO_2_ reduction by a glassy carbon electrode immersed only in AgNO_3(aq)_ and subsequently rinsed with water was also evaluated as a control experiment (green line).

Ag complexes containing N-based heterocycles form one class of molecular CO_2_ reduction catalysts (Tornow et al., 2012). Therefore, a Ag triazole catalyst was synthesized by soaking a glassy carbon electrode modified with the amino-terminated triazole in a solution of AgNO_3_ (Figure 2, green, R_1_ = –H). A LSV of the Ag triazole complex in the presence of CO_2_ displays a peak around −1.5 V and a onset potential of about −1.1 V, indicating that the complex electrocatalytically reduces CO_2_ (Figure 7, black line). Control experiments performed using a bare glassy carbon electrode, an electrode only immersed in AgNO_3_, or an electrode modified with only the triazole ligand do not exhibit this peak and have a more negative onset potential of about −1.25 V (Figure 7, blue, green, and red lines, respectively). These experiments demonstrate that the Ag triazole complex is a more effective CO_2_ reduction catalyst than any of its individual components.

**Figure 8:**
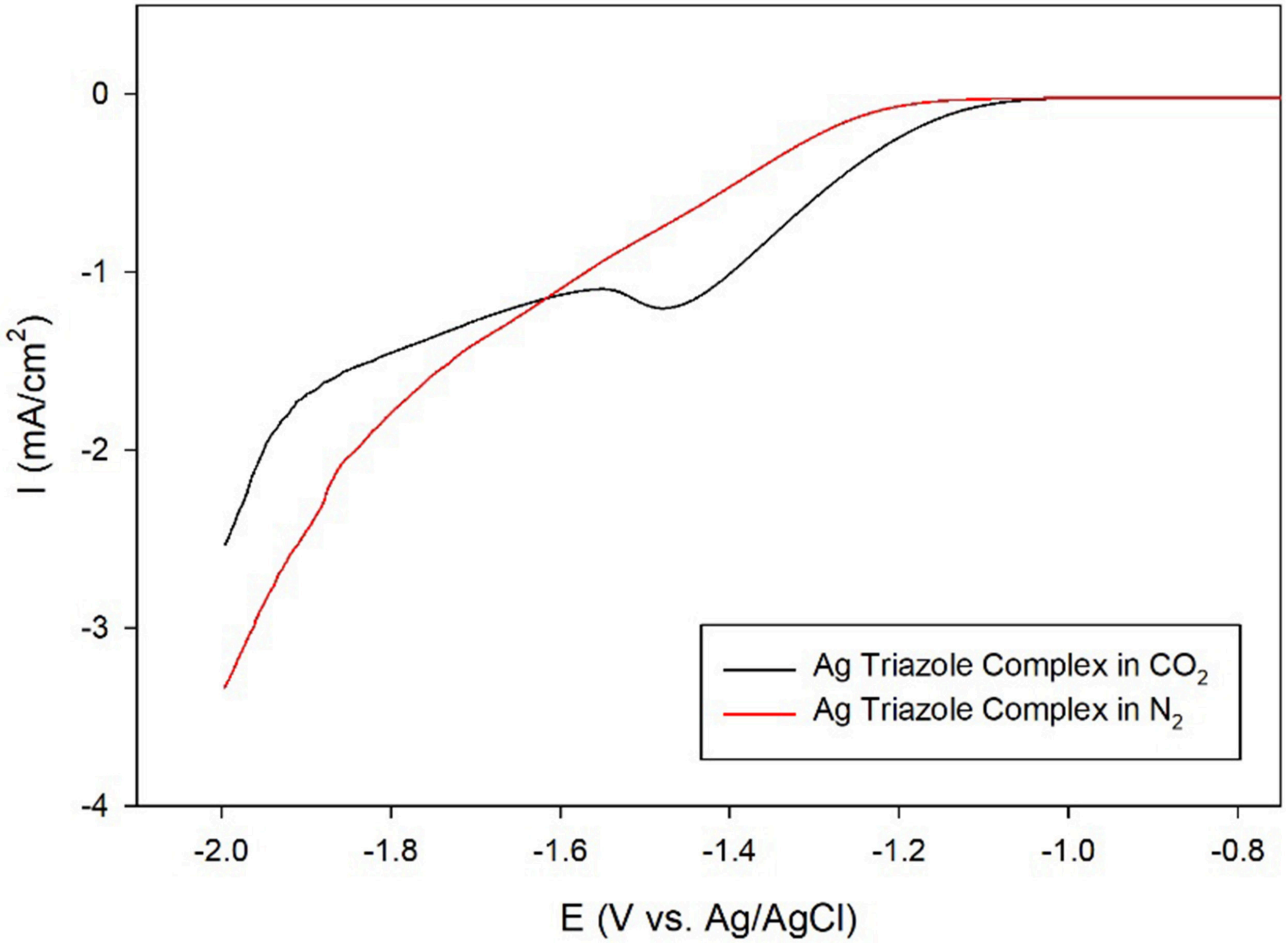
Linear sweep voltammograms by a glassy carbon electrode modified with the Ag complex of the amino-terminated triazole in CO_2_-saturated (black line) and N_2_-saturated (red line) pH 7 phosphate buffer at a scan rate of 10 mV/s.

A further control experiment of a LSV of the Ag triazole complex conducted in a N_2_ environment shows a similar onset potential of about −1.25 V and also does not exhibit a peak at −1.5 V (Figure 8, red line). This experiment provides two important insights into the catalytic behavior of these systems. First, the lack of a peak in the LSV under N_2_ further confirms that the Ag triazole complex catalyzes CO_2_ reduction. Second, the similarity of the LSV of the Ag triazole complex in N_2_ to the other control experiments presented in Figure 7 suggests that the cathodic current observed in these cases is due to the H_2_ evolution reaction. In other words, a bare glassy carbon electrode or an electrode modified with only the triazole ligand does not significantly reduce CO_2_ under these conditions.

**Figure 9:**
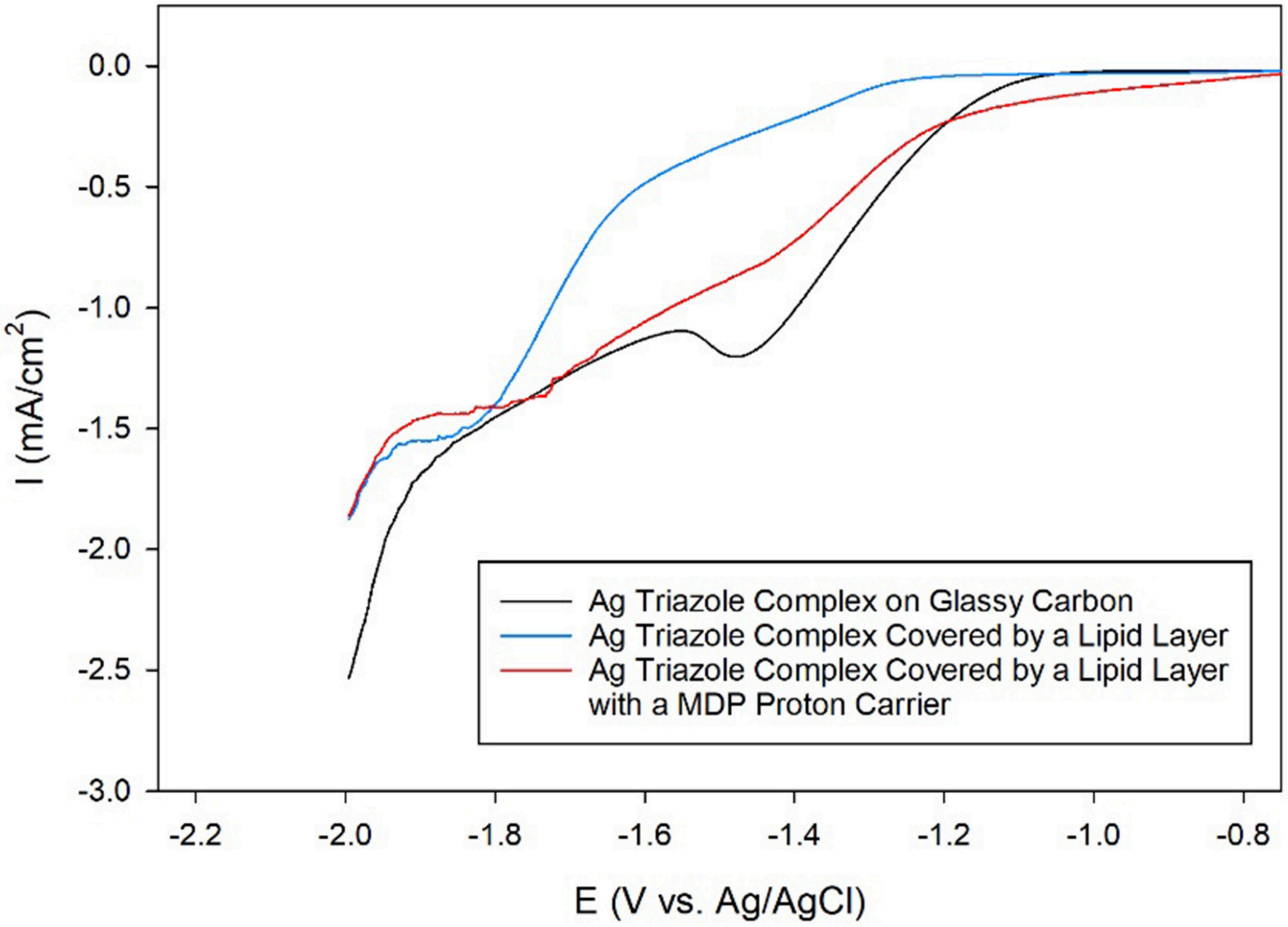
Linear sweep voltammograms of CO_2_ reduction by a glassy carbon electrode modified with the Ag complex of the amino-terminated triazole (black line) covered by a lipid membrane (blue line) with MDP proton carrier (red line) in pH 7 phosphate buffer at a scan rate of 10 mV/s.

Having established that the Ag triazole complex catalyzes CO_2_ reduction, its catalytic performance was next measured using a membrane-modified electrode. When the catalyst is covered by a lipid monolayer (Figure 2, blue, R_1_ = –H), the onset potential for catalysis shifts negative and the CO_2_ reduction peak is not present (Figure 9, blue line), indicating that CO_2_ reduction is significantly inhibited in this case. The lipid impedes access of protons to the catalyst, which are necessary for most CO_2_ reduction processes (Costentin et al., 2013). Furthermore, the current density in the LSV with lipid only reaches about −1.9 mA/cm^2^ at −2.0 V as compared to about −3.4 mA/cm^2^ at −2.0 V for the Ag triazole complex without lipid in N_2_ (Figure 8, red line). Since the Ag triazole complex without lipid in N_2_ catalyzes the H_2_ evolution reaction as discussed in the preceding paragraph, the decrease in the magnitude of current density observed for the lipid-modified catalyst indicates that the H_2_ evolution reaction is suppressed. This finding is consistent with the idea that the lipid layer slows down proton transfer to the catalyst since the H_2_ evolution reaction requires protons.

The addition of a proton carrier, either an alkyl phosphate, MDP, or an alkyl boronic acid, DBA, into the lipid layer (Figure 2, red, R_1_ = –H) produces significant changes in the voltammetry of the Ag catalyst (Figure 9, red line and Figure S5, red line). For both proton carriers, there is more catalytic current in the regime of −1.2 to −1.8 V compared to the lipid-only case, and there are not significant differences in the integrity of the lipid layer as determined by blocking experiments (Figure S6). These results suggest that, compared to the lipid-only case, the activity of the CO_2_ reduction catalyst is altered by the presence of the proton carrier, likely due to the enhancement of proton transfer rates to the catalytic active site. The amount of current enhancement observed depends upon the quantity of proton carrier added to the lipid layer (Figure S7), which dictates the rate of proton transfer to the catalyst. The LSVs of the Ag catalyst in the presence of proton carrier also depend upon temperature with greater current densities for CO_2_ reduction observed as the temperature is increased (Figure S8).

**Figure 10:**
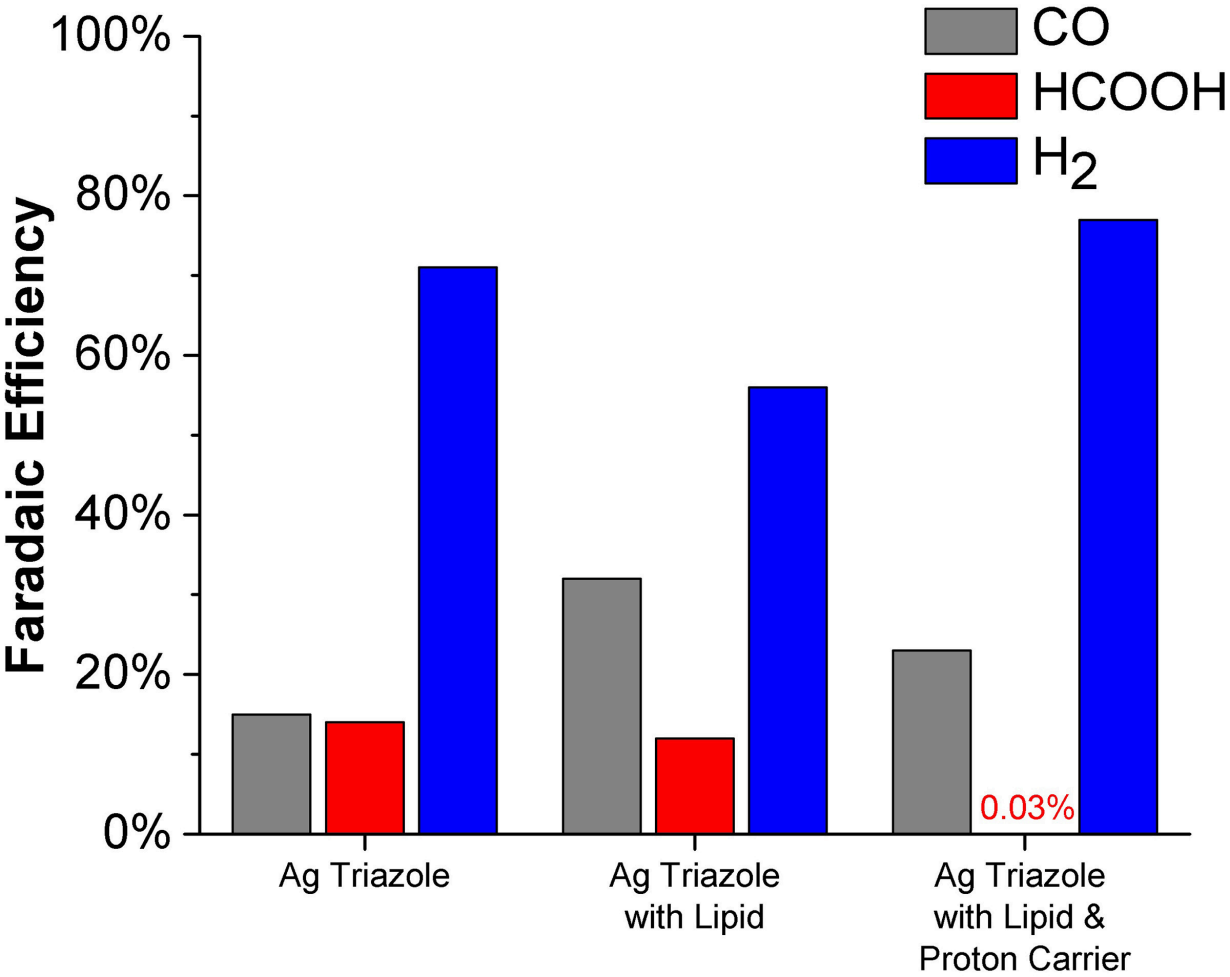
Faradaic efficiencies for CO (gray), HCOOH (red), and H_2_ (blue) production from the Ag triazole complex (left) with lipid (middle) and DBA proton carrier (right) obtained from chronoamperometry experiments at −1.75 V vs. Ag/AgCl.

The CO_2_ reduction products obtained using the Ag triazole catalyst in different electrode environments at −1.75 V were quantified (Figure 10). The unmodified Ag triazole complex on glassy carbon produces nearly equal amounts of CO and HCOOH (~15% Faradaic efficiency each) along with substantial quantities of H_2_. These results confirm that the Ag triazole complex is an active CO_2_ reduction catalyst. However, the catalyst also produces a larger amount of H_2_ than is observed with Nafion-bound Ag triazole complexes (Tornow et al., 2012). The greater quantity of H_2_ produced likely originates from exposed portions of the carbon surface that are not modified by the Ag triazole monolayer.

Covering the Ag triazole catalyst with a lipid layer decreases the Faradaic efficiency of H_2_ production from ~71 to ~56%. The decreased quantity of H_2_ produced is attributed to the hydrophobic nature of the lipid environment, which decreases the rate of proton transfer to the catalyst. With an impeded proton transfer rate, the catalyst has more time to bind and reduce CO_2_ to either CO or HCOOH. This alteration in mechanism with a change in proton transfer rate is displayed schematically in Figure S9. The results indicate that the catalyst preferentially reduces CO_2_ to CO (~32%) over HCOOH (~12%). Again, the hydrophobic nature of the lipid environment likely dictates product selectivity. Water is formed as a coproduct with CO, but not with HCOOH. We hypothesize that the preference for CO formation is due to the favorable elimination of water out of the hydrophobic lipid interior, which shifts the reaction equilibria toward CO production.

The product selectively is further altered when a proton carrier is incorporated in the lipid layer. Specifically, the Faradaic efficiency for H_2_ increases from ~56 to ~77% upon adding the proton carrier. The proton carrier increases proton transfer kinetics to the catalyst, which favors the production of H_2_. Interestingly, the proton carrier drastically increases the ratio of CO to HCOOH generated and almost completely eliminates HCOOH production (~0.03% Faradaic efficiency). The exact origin of this change in product selectivity is unknown, but possibly originates from interactions between the proton carrier and CO_2_ reduction intermediates. The CO_2_ reduction products of this system were also quantified as a function of temperature (Figure S10). Decreasing the reaction temperature to 1°C increases the Faradaic efficiency for HCOOH production to ~3%. At this low temperature, previous studies have demonstrated that proton carriers cannot undergo flip-flop diffusion because the lipid layer is below its gel-phase transition temperature (Barile et al., 2014). Therefore, we anticipate that at 1°C, the Ag catalyst behaves as if there is no proton carrier. This hypothesis is supported by the observation that the ratio of CO to HCOOH production with proton carrier in the cold is similar to the lipid-only case at room temperature.

Lastly, the effect of voltage on the CO_2_ product speciation was tested. The CO_2_ products generated at −2 V are displayed in Figure S11. At this higher overpotential, the Ag triazole catalyst in the absence of lipid produces similar quantities of CO, HCOOH, and H_2_ as compared to the −1.75 V case. However, when the catalyst contains a lipid layer with or without the proton carrier, the Faradaic efficiencies for CO and HCOOH both decrease to ~2%, suggesting that the lipid layer inhibits CO_2_ reduction at this higher overpotential.

## Conclusions

We designed membrane-modified electrodes containing metal triazole complexes that electrocatalyze the reduction of O_2_ and CO_2_. For the O_2_ reduction reaction, the complexes were anchored using SAMs on both Au and glassy carbon electrodes. By covering the catalysts in a lipid layer containing proton carriers, the kinetics of proton transfer to the complexes can be controlled on both substrates. The membrane-modified electrocatalytic systems developed on glassy carbon electrodes have a wider electrochemical window than those using Au, which enable the study of CO_2_ reduction by lipid-covered catalysts. The results suggest that the relative rates of H_2_, CO, and HCOOH production can be altered through the use of membranes.

## Author contributions

SS performed experiments. Both SS and CB designed experiments, interpreted the data, and wrote the paper.

### Conflict of interest statement

The authors declare that the research was conducted in the absence of any commercial or financial relationships that could be construed as a potential conflict of interest.
