# Supplementary material for: Membrane-Modified Metal Triazole Complexes for the Electrocatalytic Reduction of Oxygen and Carbon Dioxide
Source: Front Chem. 2018 Nov 6;6:543. doi: 10.3389/fchem.2018.00543 (PMC6232292; doi:10.3389/fchem.2018.00543)
Supplement: Supplementary file 1 [file Data_Sheet_1.pdf]

Supplementary Material

Membrane-Modified Metal Triazole Complexes for the Electrocatalytic Reduction of Oxygen and Carbon Dioxide

Skye N. Supakul, Christopher J. Barile*

*** Correspondence:**

Christopher Barile

cbarile@unr.edu

Supplementary Figures

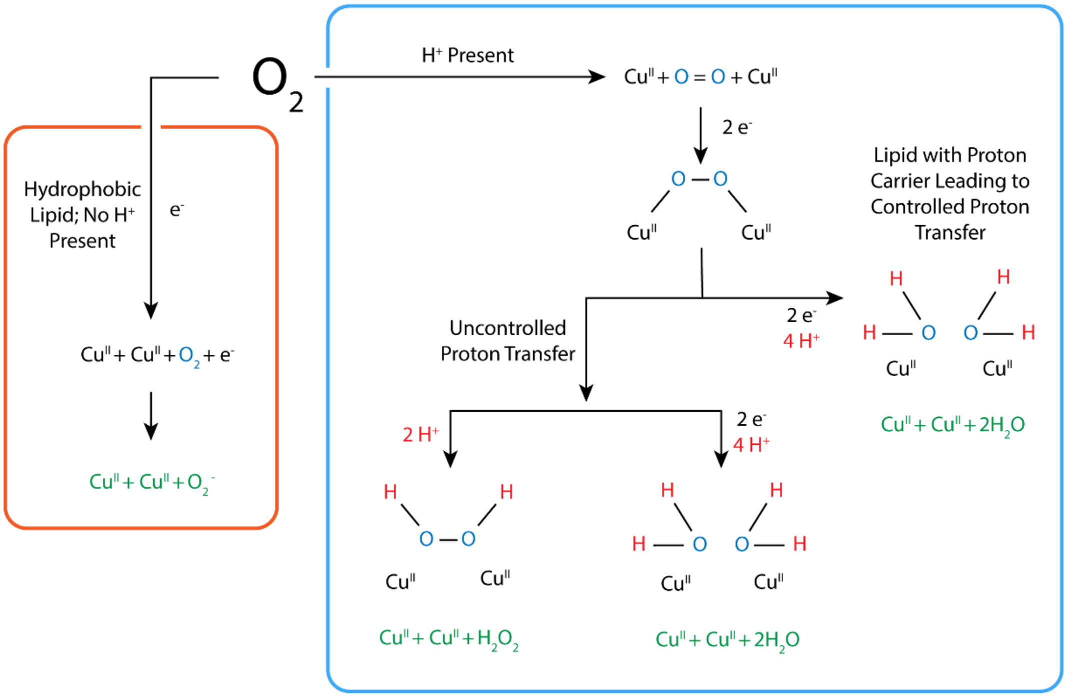

Figure S1: Schematic of mechanistic pathways for the ORR in different electrode environments.

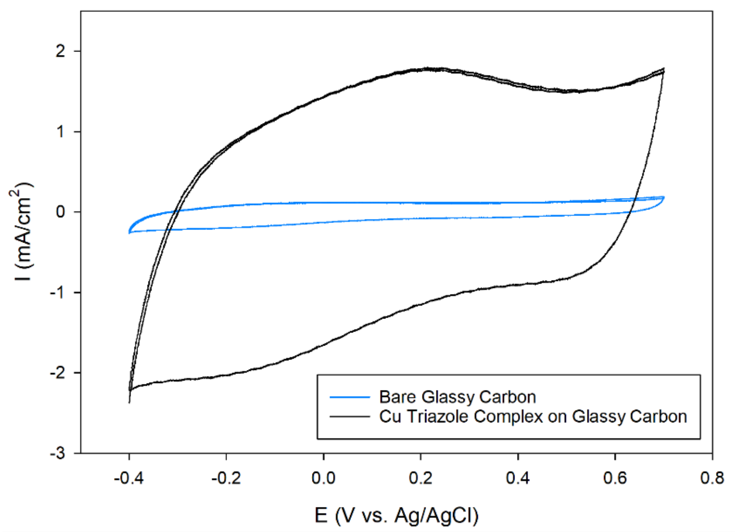

Figure S2: Cyclic voltammograms of a glassy carbon electrode (blue line) modified with the Cu complex of the amino-terminated triazole (black line) in pH 7 phosphate buffer under N_2 at a scan rate of 2000 mV/s.

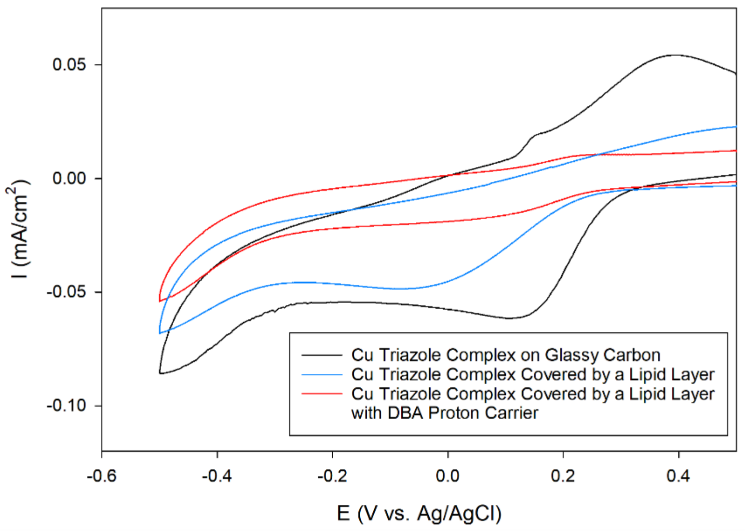

Figure S3: Cyclic voltammograms of a glassy carbon electrode modified with the Cu complex of the amino-terminated triazole (black line) covered by a lipid membrane (blue line) with DBA proton carrier (red line) in 1.5 mM $K_3Fe(CN)_6$ and 100 mM NaCl at a scan rate of 50 mV/s.

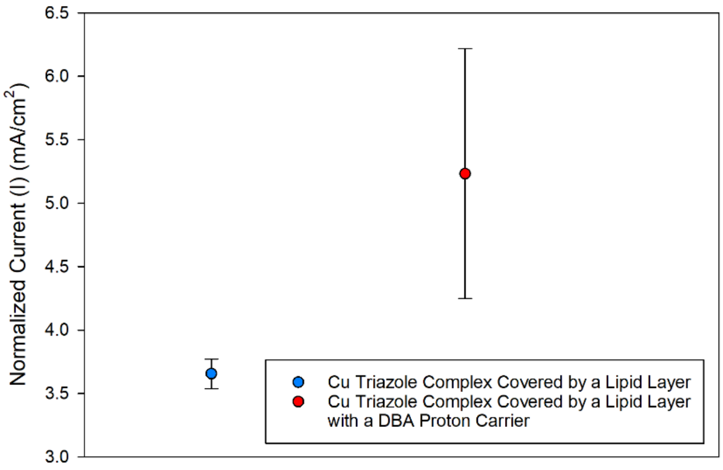

Figure S4: Comparison of normalized peak current densities of the Cu complex of the amino-terminated triazole covered by a lipid membrane with (red point) and without (blue point) DBA proton carrier. For each datum point, the peak current densities were averaged over four different experimental trials. For each trial, the peak current density was divided by the integrated area of the cathodic wave of the blocking experiment performed in Figure S2. This analysis provides a measure for how much catalytic current density is produced relative to a quantitative metric reflecting the integrity of the lipid layer. On average, the magnitude of the peak catalytic current density normalized in this manner is about 40% greater when the proton carrier is present in the lipid layer. This result indicates that the enhancement of catalytic current observed in the voltammetry with the proton carrier is due to enhanced reaction kinetics and is not simply caused by changes in lipid monolayer integrity.

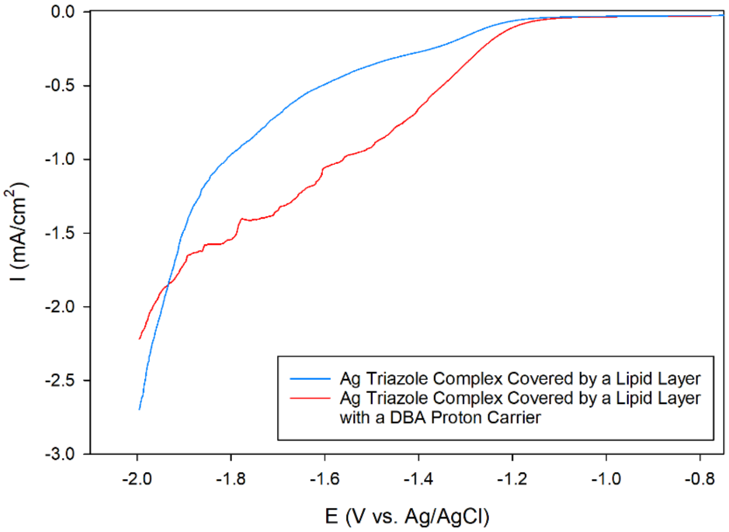

Figure S5: Linear sweep voltammograms of CO_2 reduction by a glassy carbon electrode modified with the Ag complex of the amino-terminated triazole covered by a lipid membrane (blue line) with DBA proton carrier (red line) in pH 7 phosphate buffer at a scan rate of 10 mV/s.

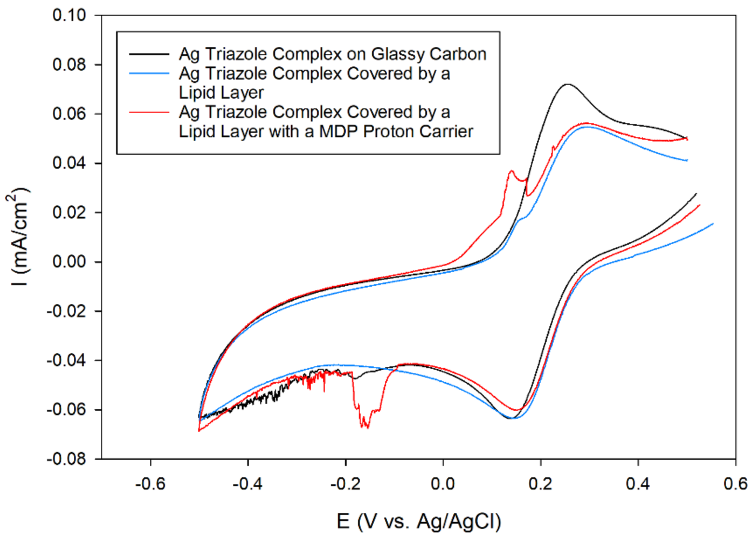

Figure S6: Cyclic voltammograms of a glassy carbon electrode modified with the Ag complex of the amino-terminated triazole (black line) covered by a lipid membrane (blue line) with MDP proton carrier (red line) in 1.5 mM $K_3Fe(CN)_6$ and 100 mM NaCl at a scan rate of 50 mV/s.

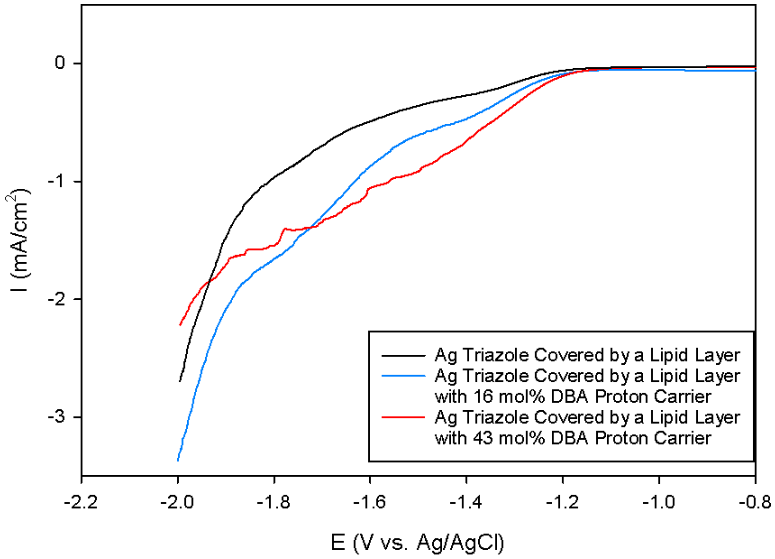

Figure S7: Linear sweep voltammograms of CO_2 reduction by a glassy carbon electrode modified with the Ag complex of the amino-terminated triazole (black line) covered by a lipid membrane with 43 mol% DBA (red line) and 16 mol proton carrier (red line) in pH 7 phosphate buffer at a scan rate of 10 mV/s.

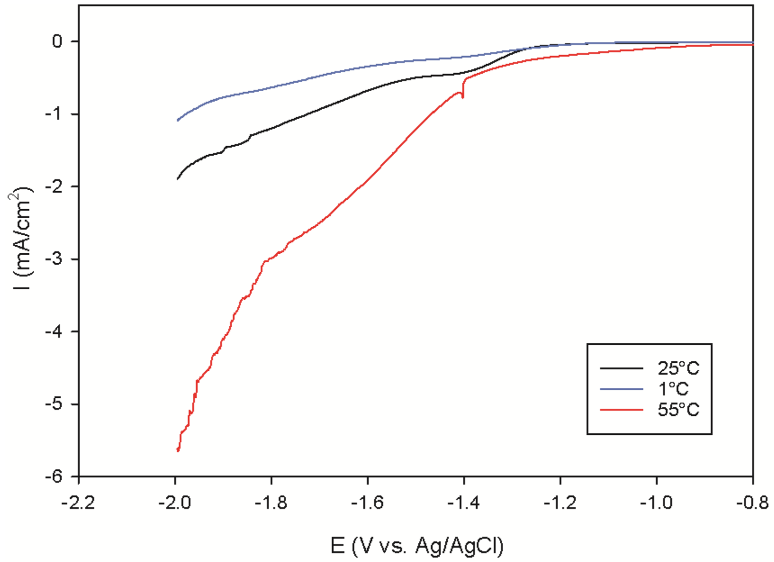

Figure S8: Linear sweep voltammograms of CO_2 reduction by a glassy carbon electrode modified with the Ag complex of the amino-terminated triazole covered by a lipid membrane with DBA proton carrier in pH 7 phosphate buffer at a scan rate of 10 mV/s at 1°C (blue line), 25°C (black line), and 55°C (red line).

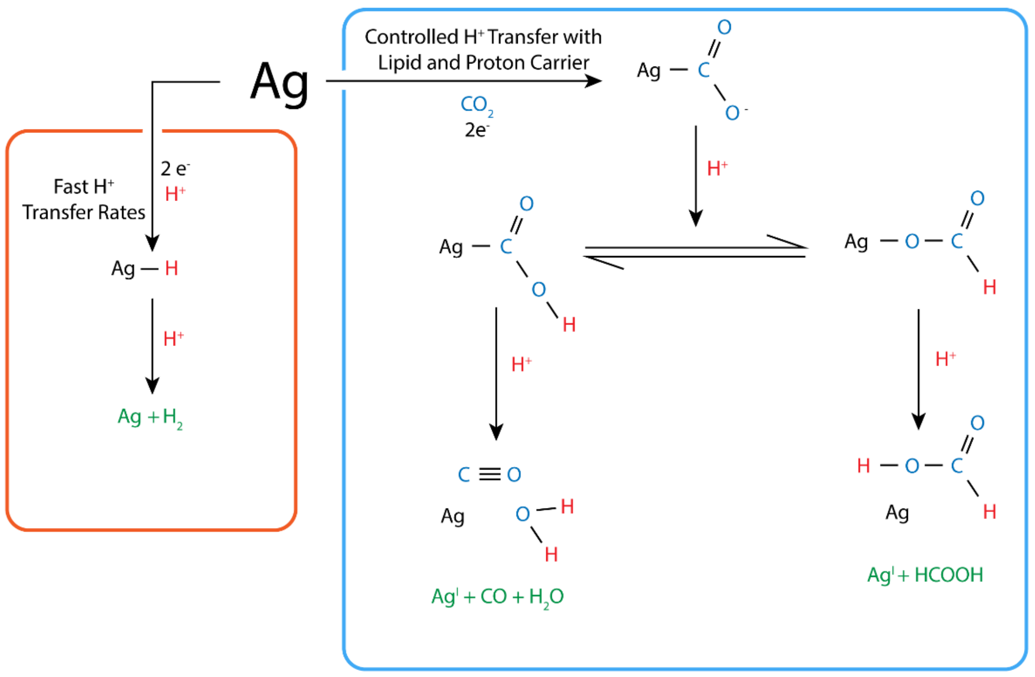

Figure S9: Schematic of possible mechanistic pathways for the CO_2 with varying proton transfer kinetics.

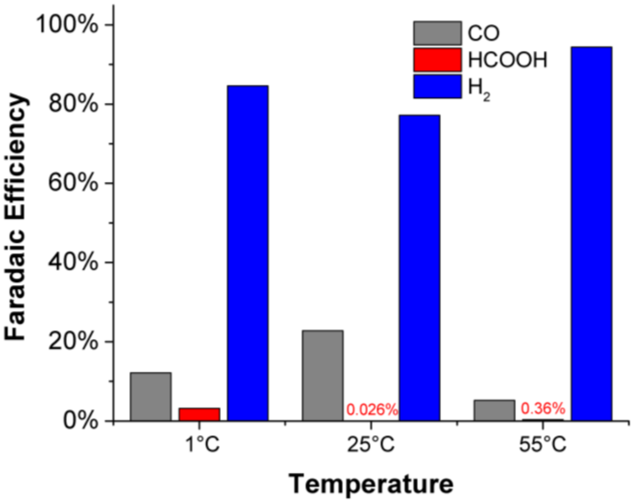

Figure S10: Faradaic efficiencies for CO (grey), HCOOH (red), and H_2 (blue) production from the Ag triazole complex with lipid and DBA proton carrier obtained from chronoamperometry experiments at -1.75 V vs. Ag/AgCl at three different temperatures.

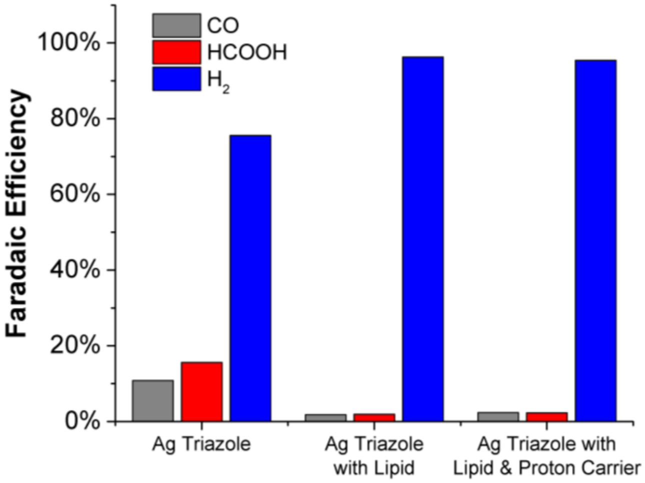

Figure S11: Faradaic efficiencies for CO (grey), HCOOH (red), and H_2 (blue) production from the Ag triazole complex (left) with lipid (middle) and DBA proton carrier (right) obtained from chronoamperometry experiments at -2 V vs. Ag/AgCl.
